# Magnetic ordering temperature of nanocrystalline Gd: enhancement of magnetic interactions via hydrogenation-induced “negative” pressure

**DOI:** 10.1038/srep22553

**Published:** 2016-03-02

**Authors:** E. A. Tereshina, S. Khmelevskyi, G. Politova, T. Kaminskaya, H. Drulis, I. S. Tereshina

**Affiliations:** 1 Institute of Physics CAS, 18221 Prague, Czech Republic; 2 Center for Computational Materials Science, Vienna University of Technology, A-1060 Vienna, Austria; 3 Baikov Institute of Metallurgy and Materials Science RAS, 119991 Moscow, Russia; 4 Faculty of Physics, M. V. Lomonosov Moscow State University, 119991 Moscow, Russia; 5 Institute of Low Temperature and Structure Research PAS, 50-950 Wroclaw, Poland; 6 International Laboratory of High Magnetic Fields and Low Temperatures PAS, 53-421 Wroclaw, Poland

## Abstract

Gadolinium is a nearly ideal soft-magnetic material. However, one cannot take advantage of its properties at temperatures higher than the room temperature where Gd loses the ferromagnetic ordering. By using high-purity bulk samples with grains ~200 nm in size, we present proof-of-concept measurements of an increased Curie point (*T*_C_) and spontaneous magnetization in Gd due to hydrogenation. From first-principles we explain increase of *T*_C_ in pure Gd due to the addition of hydrogen. We show that the interplay of the characteristic features in the electronic structure of the conduction band at the Fermi level in the high-temperature paramagnetic phase of Gd and “negative” pressure exerted by hydrogen are responsible for the observed effect.

Gadolinium, a rare earth magnet with a very high saturation polarization originating from the localized half-filled 4*f*-electronic shell is often regarded as an almost ideal soft-ferromagnetic material due to the small magnetic anisotropy^1^,^2^,^3^. Apt for near-room-temperature magnetic refrigeration^4^,^5^,^6^,^7^,^8^, the Curie temperature (*T*_C_) of Gd hinders its application in other technological fields which require a soft magnet operating at higher temperatures. Interatomic distances affect the strength of magnetic interactions and an increase of *T*_C_ under pressure has been predicted^9^,^10^. However, experimental evidence obtained on a Gd crystal under hydrostatic pressure disagrees on the direction of the Curie point shift^11^. Therefore, a series of desirable samples can be produced by boosting the volume of Gd.

Volume expansion can be created by absorption of light elements, in particular, hydrogen. Electron bonding of hydrogen with electropositive Gd competes with a “negative” pressure exerted by hydrogenation. This may reduce the strength of the RKKY interaction and lower the *T*_C_. Gd hydrided to low hydrogen concentrations by forming an α-GdH_*x*_ (*hcp)* solid solution preserves ferromagnetism^12^. Further increasing the hydrogen content causes sequential phase transitions into antiferromagnetic β- (*fcc*, GdH_2_) and γ-phases (*hcp*, GdH_3_) with the ordering temperatures ~20 and 4 K, respectively. Several previous attempts to observe a hydrogen-induced Curie temperature change in coarse-grained Gd and thin films were unsuccessful^12^,^13^,^14^. This is probably due to several reasons, including sample purity^15^ and finite size effects (for thin films). The latter are known to reduce^16^,^17^,^18^,^19^ the *T*_C_ significantly compared to the coarse-grained and bulk Gd. A promising result with an increased *T*_C_ was reported recently for the structurally inhomogenous α-GdH_*x*_^20^ consisting of needle-shaped micro-crystals embedded in nano-grained Gd matrix.

Here, we report an advance in the experimental and theoretical determination of the hydrogen-induced change of Curie temperature in Gd. We use high-purity bulk nanocrystalline, homogenous samples of Gd prepared by vacuum distillation^21^ with grains ~200 nm in size, which is then artificially doped with hydrogen. Importantly, the grains size in the studied material is not critical for the magnetic properties of Gd^22^. The Arrott-Belov plot method used for determining the *T*_C_s makes it easy to overcome unwanted thermal and magnetic field effects^23^. Another method of finding the *T*_C_ is from the magnetocaloric effect (MCE) measurements. The former enables the true *T*_C_ in pure and hydrogen-charged Gd to be determined, offering a direct comparison with first principle calculations. The main purpose of the work is the determination of the physical mechanisms behind the hydrogen-induced Curie temperature enhancement in Gd.

## Experimental Results and Discussion

**Figure 1:**
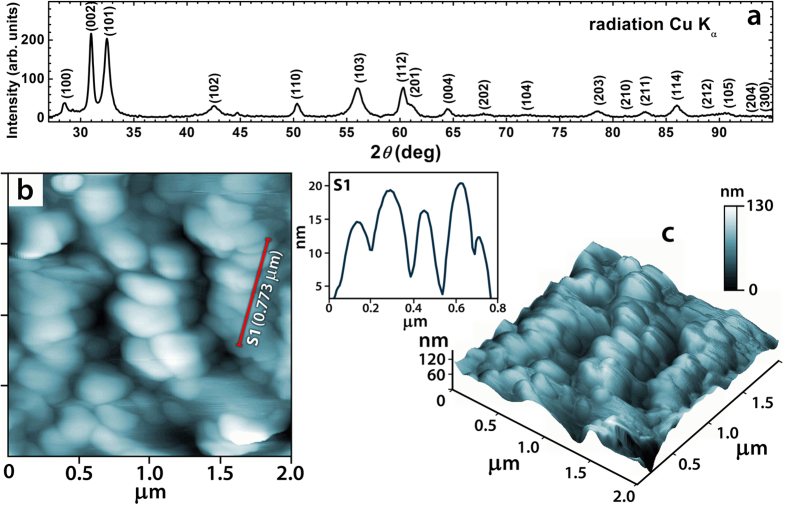
Room-temperature x-ray diffraction pattern of Gd (**a**). Typical AFM surface topography (**b**). Inset: (*S1*) cross-sectional analysis performed along the line *S1* in Fig. 1 (**b**). Corresponding 3D surface image for distilled Gd (**c**).

Elemental analysis of Gd revealed the 99.99 wt.% purity with respect to other rare earth elements. The interstitial contents were determined as follows: 56 wt. ppm of C, 1 wt. ppm of N and 306 wt. ppm of O. (This is comparable or better than the sample purities in refs 24 and 25). Figure 1a shows a room-temperature x-ray diffraction pattern of the high-purity Gd. The distilled sample has an *hcp* single-phase structure and its structural characteristics (Table 1) agree well with the literature^4^,^8^. The AFM (surface morphology) image obtained for this sample (piece) reveals the presence of mounds consisting of nanosized grains (Fig. 1b). The 3D-structural morphology is shown in Fig. 1c. The metal precipitation from the gaseous phase resulted in the formation of an undulating surface. The results of cross-sectional analysis performed along the line (*S1*) shown in Fig. 1b are presented in Fig. 1(*S1*). The average grain size was estimated to be 200 nm. The height difference between mounds is ranging between 20 and 80 nm.

The samples remained monolithic after exposure to hydrogen. Grains in Gd-H_*x*_ increased in size to 400–500 nm as a result of heating during the hydrogenation procedure. Yayama and Tomokiyo claimed^12^ that Gd α-hydrides can be formed up to hydrogen concentrations 0.35 at.H/f.u. However, both our study and available literature data^14^ show that it is rather difficult to avoid formation of the parasitic dihydride GdH_2_ phase (β-phase) when preparing α-hydrides. Nevertheless, we succeeded in obtaining several single-phase α-GdH_*x*_ samples by optimizing (minimizing) the time of the hydrogenation procedure. The maximum hydrogen content in α-GdH_*x*_ reached 0.22±0.02 at.H/f.u. (see the structural characteristics of α-GdH_0.22_ in Table 1). The structural studies of parent and hydrided samples were carried out under similar conditions so that the increase of the unit cell volume Δ*V/V* ~1% upon hydrogenation has been found for α-GdH_0.22_. We also obtained samples with higher hydrogen concentrations of up to 1 at.H/f.u. These, however, contained traces of the β-phase.

**Figure 2:**
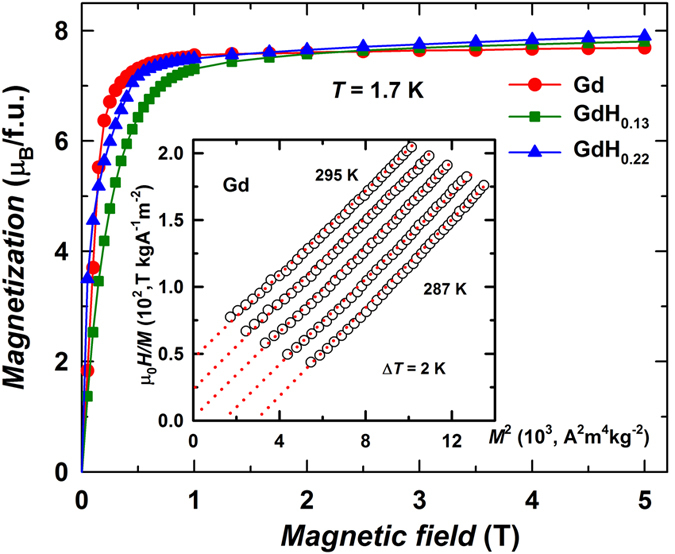
Field dependencies of magnetization of Gd, α-GdH_0.13_ and α-GdH_0.22_ at 1.7 K. The inset: experimental Arrott-Belov curves for Gd in the vicinity of *T*_C_.

Figure 2 shows the *M* vs. *H* curves for Gd and α-GdH_*x*_ in fields up to 5 T at 1.7 K. A small increase of spontaneous magnetization *M*_s_ observed in α-GdH_*x*_ is in line with theoretical calculations presented below. Note that most reports on lattice-expanded Gd are on thin-film specimen, where the level of control (regarding the quantity of expansion) is rather limited. For thin Gd films, the saturation magnetization was found to drop with increasing unit cell size^1^ (strained *hcp* phase) or when obtaining Gd in other structural modifications (*fcc*)^26^. For a hydrogenated bulk specimen with rather large grains, strain and structural factors of thin films leading to the *M*_s_ decrease are eliminated.

**Figure 3:**
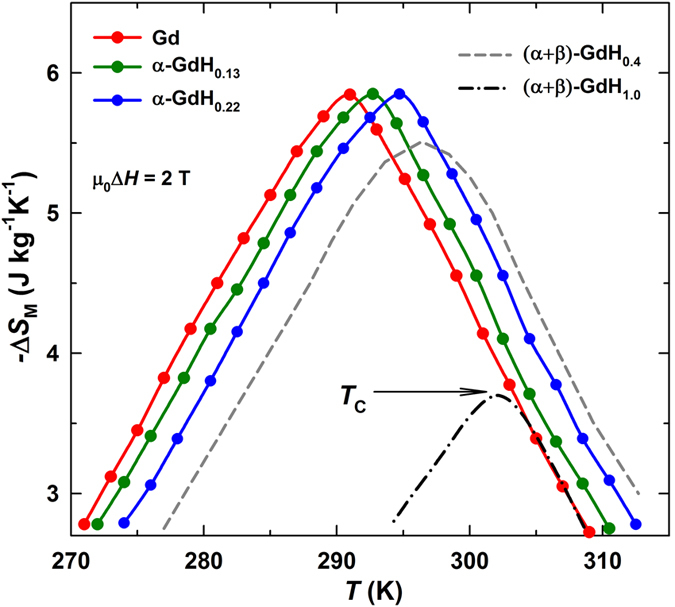
Magnetic entropy change Δ*S*_M_ vs. temperature for Gd, α-GdH_0.13_ and α-GdH_0.22_ at a field change of 2 T. The data for (α + β)-GdH_0.4_ and (α + β)-GdH_1.0_ are shown for comparison.

The Curie temperatures of Gd and α-GdH_0.22_ were determined from the Arrott-Belov plots (inset in Fig. 2) as 290.8 ± 0.2 K and 296.1 ± 0.2 K, respectively (Table 1). Another method of finding the *T*_C_ is by following the maximum of the magnetocaloric effect (MCE). The magnetic entropy change, Δ*S*_mag_, corresponding to the magnetic field change from zero to 2 T was calculated from the magnetization isotherms using the Maxwell relation for parent Gd and Gd-H_*x*_ (Fig. 3). This method defines the *T*_C_ in Gd and α-GdH_0.22_ as 291.0 ± 0.5 K and 295.0 ± 0.5 K, respectively. The highest observed *T*_C_ is 302.0 ± 0.5 K in (α + β)-GdH_1.0_. Importantly, the maximum MCE in pure Gd is 5.84 J/kg·K. This is comparable with that of a Gd single crystal^4^,^24^. The MCE of α-GdH_*x*_ remains of the same order of magnitude as in parent Gd while MCE in (α + β)-GdH_*x*_ gradually decreases due to the increasing content (21 and 50% for *x* = 0.4 and 1.0, respectively) of the non-magnetic at this temperature β-phase.

For Gd (and other rare earths) finite-size effects become critical upon lowering dimensions below 10 nm^22^. The *T*_C_ of nanostructured Gd prepared by a method similar to ours begins to decrease for crystallites smaller than 140 nm^16^. We find that *T*_C_ of distilled Gd is similar to single crystals^24^,^27^. It stems from rather large grains ~200 nm, making the impact of finite size effects negligible. As reviewed by Dan’kov *et al*.^24^, experimentally observed *T*_C_s may vary depending on the purity of the studied single crystal and on the method used to determine the *T*_C_. Belov-Arrott plots used to locate *T*_C_ from *M*(*H*) data allow us to determine unambiguously the increase of *T*_C_ upon hydriding high purity Gd.

## Theoretical analysis and conclusions

In order to explore the physical mechanisms behind the hydrogen-induced Curie temperature enhancement in Gd, we performed first-principle calculations. Magnetic exchange constants of the Heisenberg type Hamiltonian were found using a magnetic force theorem^28^ embedded^29^ in Korringa-Kohn-Rostokker band structure method^30^ within the framework of the Local Spin Density Approximation^31^. In our calculations, the *c*/*a* ratio of the *hcp* structure was fixed at values of experimental Gd and GdH_0.22_ (Table 1) while the unit cell volume was varied in order to mimic the effect of various negative chemical pressure induced by hydrogenation. The approach used for calculation is similar to that of ref. 32 performed for pure Gd in fixed lattice geometry: spin-polarized open core treatment of the Gd 4*f*-states and an *spd*-basis for the conduction band. The *spd*-valence band was calculated using a basis function expansion up to *l*_max_ = 3 and an Atomic Sphere Approximation. The band structure calculations where converged with 14950 *k*-points and a convergence of exchange constants was achieved by integrating over 341595 *k*-points in the full Brillouine zone of a hexagonal lattice.

The local character of the 4*f*-electron moments, the very small magnetic anisotropy as well as crystal field effects due to the half-filled 4*f*-shell (zero total angular momentum) make pure *hcp* Gd a good Heisenberg system. To calculate exchange constants *J*_*ij*_ of the Hamiltonian


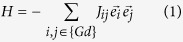


where 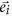 is the unit directional vector at the *i*-th Gd lattice site, the conventional Disordered Local Moment (DLM) approximation^32^ was used to model the electronic structure in the paramagnetic state. The use of DLM allows taking into account^33^ the renormalization of pair-wise exchange interactions at finite temperatures originating from the electronic structure changes in the conduction band and induced by thermal magnetic disorder of the 4*f* moments. The absolute *T*_C_ value was derived in the mean field approximation from *k*_B_*T*_C_ = 3/2 *J*_0_, where the total effective exchange constant *J*_*0*_ is a sum of *J*_*ij*_s linking a given lattice site to the remaining sites, 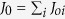.

**Figure 4:**
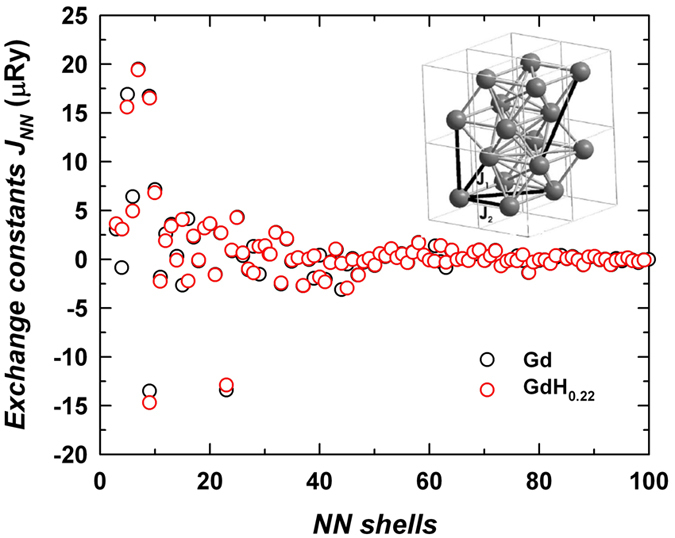
Calculated distant (starting from the third nearest neighbor) exchange interactions *J*_NN_ for Gd and GdH_0.22_. The inset: Hexagonal crystal structure of Gd and the first two nearest-neighbor exchange interactions *J*_1_ and *J*_2_.

The Curie temperatures, *T*_C_^(calc)^ of Gd and GdH_0.22_ converged to the values given in Table 1 after accounting for exchange interaction constants through up to 100 nearest-neighbor (NN) shells. Table 1 also shows local conduction band moments, *m*, induced at the Gd atomic sites. (Note that in addition, Gd^3+^ ion has a localized moment of 7 μ_B_). Two of the strongest NN interactions, inter-plane *J*_1_ and in-plane *J*_*2*_ exchange constants (inset in Fig. 4), are also shown in Table 1. The values differ from each other due to deviations from an ideal *hcp* lattice. The remaining exchange constants *J*_NN_ (Fig. 4) are an order of magnitude smaller.

**Figure 5:**
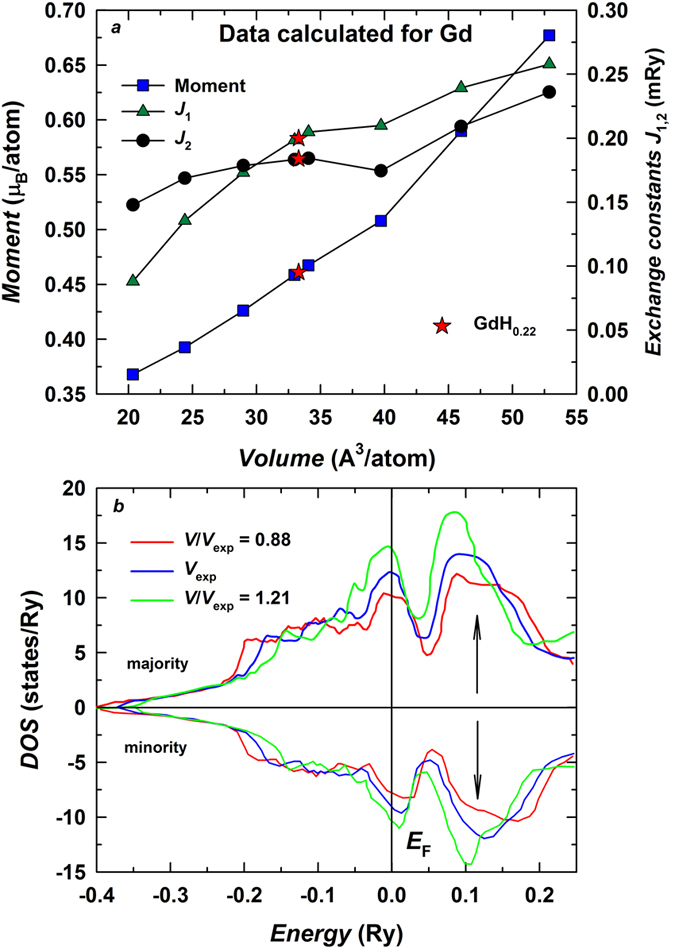
(**a**) The unit cell volume variation of conduction band moments and *J*_1_ and *J*_2_ exchange interactions in Gd and GdH_0.22_. (**b**) DOS for the majority and minority spin-channels of Gd conduction band in the paramagnetic state obtained by DLM approximation using three different volumes *V*/*V*_exp_.

The difference between the *T*_C_s of pure Gd and GdH_0.22_, 4.5 K, is in excellent agreement with experimental data. Remarkably, the overall increase of *T*_C_ is caused by long-distance interactions beyond the 20NN shells, while the first 20NN shells give less than a 1 K increase. The increase of exchange interactions due to volume expansion may look contra-intuitive when viewing the magnetic interactions in pure Gd entirely in terms of a simple RKKY model, in which the volume expansion driving the atoms farther may reduce the interaction strength. However, the *T*_C_ change due to the volume increase can be readily understood by examining changes in the electronic structure of the Gd conduction band. To clarify this point, we expose the results of DLM calculations performed for different unit cell volumes in Fig. 5a. We present exemplary results for the *c*/*a* ratio fixed at the experimental value for pure Gd since the calculation performed for the experimental *c*/*a* ratio of GdH_0.22_ does not produce any visible changes in the figure. As the unit cell of Gd expands, the induced conduction band moments in the paramagnetic state increase. This leads to the increase of the Hamiltonian’s exchange constants (Eq. 1). However, the moment’s increase is not the only volume-related effect which causes enhancement of the distant interactions^2^. Another consequence of the volume expansion is the increased Density of States (DOS) of the conduction band at the Fermi level *E*_F_ (see Fig. 5b for different volumes *V*/*V*_exp_ with the experimental *V*_exp_ value for parent Gd). Since the long distance magnetic interaction between the 4*f* moments is mediated by conduction electrons, the increase of DOS at *E*_F_ increases the *T*_C_.

Photoemission experiments^34^,^35^ showed that the conduction band of Gd retains local spin-polarization in the paramagnetic state, and the situation is far from a simple rigid-band Stoner-like picture. This feature can be promptly captured using a DLM approximation for pure Gd. Our calculations reveal two major competing forces in the paramagnetic state, namely, local spin-polarization of the conduction band arising due to the exchange interaction with the localized 4*f* shell and the band effects resulting from hybridization with neighboring sites. The latter tend to reduce spin-splitting of the majority and minority spin channels^36^. The effect is demonstrated in Fig. 5b. Spin-splitting of the majority and minority spin-channels is indeed small, and nearly all local spin polarization is due to redistribution of spectral weight caused by the exchange interaction with the local 4*f*-moments. Hybridization decreases as the volume increases, and the conduction band becomes narrower (Fig. 5b). Both DOS at the Fermi level and local spin-polarization of the conduction band consequently increase.

To summarize, the Curie temperature increase upon hydriding is a purely electronic effect related to the expanded Gd lattice. The latter is also responsible for the small increase of spontaneous magnetization in α-GdH_*x*_. The use of high-purity bulk parent sample with grains ~200 nm in size eliminates difficulties related to the observation of this effect in thin films. It can be suggested that, irrespective of the particular manipulation used, volume increase may grant higher efficiency to Gd as a soft magnetic material.

## Methods

### Sample preparation and characterization

The parent Gd sample was prepared by vacuum distillation into solid phase from commercially available Gd metal (GdM-1 Grade). The metal was hydrided using a Sievert-type apparatus. Hydrogen content in the samples was found volumetrically, i.e. by measuring the pressure in the hydrogenation chamber before and after the reaction. The crystal structure of the samples was characterized by x-ray diffraction (XRD). The XRD patterns were recorded at a 0.02° scanning step on a Rigaku Ultima IV diffractometer (Japan) with a Cu-*K*α radiation. The patterns were analyzed using a program PDXL by Rigaku integrated with the international database ICDD. Surface topology was investigated by an atomic force microscopy in a semi contact mode using the SMENA-A scanning electron microscope mounted on a Solver platform (NT-MDT) at ambient conditions. A high-precision silicon HA_NC probe (10 nm curvature tip radius) with an Au reflective side was used for the study (force constant 3.5 N/m and a nominal resonant frequency of 129.4 kHz).

### Magnetic study

The samples magnetization *M*(*H*) was measured on rod-shaped samples (length 5–7 mm, diameter 0.1–0.2 mm) using a PPMS-14 magnetometer (Quantum Design, USA) at a constant field step (0.05 Т) and at various temperatures (2 K step). The Curie temperatures of the parent and hydrided Gd were determined by means of the Arrott-Belov plot method based on the thermodynamic theory^23^. The accuracy of the Arrott-Belov’s method is the highest among other techniques for the *T*_C_ determination. It has a standard experimental error of ±0.2 K. The second method used to evaluate the *T*_C_ is by detecting the maximum of magnetocaloric effect. In the present work, the MCE was studied by measuring the magnetization *M*(*H*) at a field change from zero to 2 T. The magnetic entropy change Δ*S*_m_ was calculated using the Maxwell relation.

## Additional Information

**How to cite this article**: Tereshina, E. A. *et al*. Magnetic ordering temperature of nanocrystalline Gd: enhancement of magnetic interactions via hydrogenation-induced “negative” pressure. *Sci. Rep.* **6**, 22553; doi: 10.1038/srep22553 (2016).

## Figures and Tables

**Table 1:** Structural and magnetic characteristics of Gd and α-GdH_0.22_.

Material	*a*, Å	*c/a*	*V*, Å^3^	Δ*V/V*,*%*
Gd	3.633(6)	1.588(4)	65.93	–
α-GdH_0.22_	3.646(3)	1.586(2)	66.58	0.99

	m*, μ_B_/Gd	*J*_1_**, μRy	*J*_2_**, μRy	*T*_C_***^(calc)^, K	*T*_C1_****^(exp)^, K	*T*_C2_**** ^(exp)^, K
Gd	0.459	183.2	198.6	327.9	290.8(2)	291.0(5)
α-GdH_0.22_	0.461	183.9	199.9	332.4	296.1(2)	295.0(5)

^*^m is a local conduction band moment. The value of 7 μ_B_ from the localized 4*f* moment has to be added for the total moment.

^**^J_1_ and *J*_*2*_ are the inter-plane and in-plane exchange constants, respectively (cf. inset in Fig. 4).

^***^T_C1_^(exp)^ is a calculated Curie temperature.

^****^T_C1_^(exp)^ and *T*_C2_^(exp)^ are the Curie temperatures determined using Arrott-Belov plots and the MCE maximum at μ_0_Δ*H* = 2 T, respectively.
